# Exploring alternative cytokines as potential biomarkers for *Mycobacterium bovis* infection in cattle

**DOI:** 10.3389/fimmu.2026.1786944

**Published:** 2026-02-25

**Authors:** Giulia Franzoni, Federica Signorelli, Grazia Carbotti, Anna Donniacuo, Lorena Schiavo, Susanna Zinellu, Emanuela Giaconi, Pasqualino Cappuccio, Mauro Nitti, Orlando Paciello, Giuseppe Iovane, Francesco Napolitano, Maria Beatrice Boniotti, Alessandra Martucciello

**Affiliations:** 1 Department of Animal Health, Istituto Zooprofilattico Sperimentale della Sardegna, Sassari, Italy; 2 Council for Agricultural Research and Economics (CREA) - Research Centre for Animal Production and Aquaculture, Monterotondo, Italy; 3 Diagnostic Section of Lecce, Istituto Zooprofilattico Sperimentale della Puglia e della Basilicata, Foggia, Italy; 4 National Reference Centre for Hygiene and Technologies of Mediterranean Buffalo Farming and Productions, Istituto Zooprofilattico Sperimentale del Mezzogiorno, Salerno, Italy; 5 Azienda Sanitaria Locale Avellino, Avellino, Italy; 6 National Reference Centre for Bovine Tuberculosis, Istituto Zooprofilattico Sperimentale della Lombardia e dell’Emilia Romagna, Brescia, Italy

**Keywords:** biomarkers, chemokines, cytokines, IFN-γ, *Mycobacterium bovis*

## Abstract

*Mycobacterium bovis* (*M. bovis*) is the primary agent of Bovine tuberculosis (bTB) in cattle. It represents both a threat to human health and the cattle industry worldwide. Improving bTB diagnostic performance in cattle represents a key step in eradicating the disease. The interferon-gamma (IFN-γ) release (IGRA) blood assay is routinely used in the diagnosis of *M. bovis* infection, but additional cytokines might be useful as biomarkers of this infection in cattle. In our study, we evaluated the utility of sixteen immune cytokines as diagnostic biomarkers of *M. bovis* infection. Fifty-five cattle were used in this study: healthy animals (N = 19), infected (IFN-γ test positive, no post-mortem lesions; N = 17), and affected (IFN-γ test positive, visible post-mortem lesions; N = 19). Heparin blood samples were stimulated *in vitro* with bovine purified protein derivative (PPD-B), alongside controls. After 18–24 h of incubation, plasma were collected and levels of 16 key cytokines were measured: IL-1α, IL-4, IL-6, IL-10, IL-17, IL-36Ra, MIP-1α, IP-10, MCP-1, TNF, VEGF-A, IFN-γ, IL-23, IL-27, IL-35, and THBS-1. We observed that both *M. bovis* exposed cattle (both infected and affected) released higher levels of PPD-B specific IFN-γ and IP-10. On the contrary, only cattle belonging to the affected group released higher levels of PPD-B specific IL-4, IL-17, and TNF compared to healthy subjects. Canonical discriminant analyses (CDA) indicated that IP-10, IL-4, IL-17, and TNF could be useful biomarkers for infection status. In particular, our data suggest that the parallel measurement of IFN-γ and IP-10 might improve the diagnosis of *M. bovis* infection in cattle in terms of sensitivity and specificity, although this should be validated on a larger set of animals. In the CDA analysis, only a modest separation between infected and affected cattle was observed. Nevertheless, our data suggested that IL-4, IL-10, and TNF might improve, at least in part, the differentiation of cattle in diverse stages of TB infection. Overall, the data generated in our study provide a foundation to improve the diagnosis and staging of *M. bovis* in cattle.

## 1 Introduction

*Mycobacterium bovis* (*M. bovis*) is the primary agent of Bovine tuberculosis (bTB), a zoonotic disease affecting cattle and wild animals. It represents not only a threat to human health, but it also negatively affects the global cattle industry due to the negative impact on animal production and detrimental effects on animal health (1). This chronic disease is distinguished by the progressive development of the characteristic granulomas, mainly in the lungs and lymph nodes, which limits the spread of mycobacteria into the host (1, 2). The disease can disseminate within a herd before the development of obvious clinical symptoms or can stay latent for years and spread only when the animal is immunocompromised due to additional stresses or old age (1).

Control of bTB relies on early diagnosis, removal of infected animals, and tracing and containment of contact cases. Identification of infected animals is carried out with slaughterhouse surveillance (to identify animals with characteristic TB-lesions) or through ante-mortem assays, such as the intradermal tuberculin test (IDT) and the interferon-gamma (IFN-γ) release (IGRA) blood assay (1, 3, 4).

IDT measures dermal swelling primarily due to cell-mediated immune response (CMI) 72 h after intradermal injection of purified protein derivative (PPD) in the skin of tested animals (Shiller et al., 2010). The IGRA assay is instead a laboratory-based test which measures release of IFN-γ from whole blood (predominantly T lymphocytes) stimulated for 18–24 h with PPDs (5).

Nevertheless, both tests present some disadvantages: they are unable to differentiate *M. bovis* infection from bTB disease and are incapable of identifying infected animals in the first stage of infection (3, 5). Therefore, several efforts have been made to improve bTB diagnosis and understanding (6) and one of the main areas of bTB research is the discovery of new biomarkers able to early detect *M. bovis* infected animals.

Cytokines are small proteins that play a crucial role in orchestrating immune responses. They have been widely studied as biomarkers for several diseases, including tuberculosis (7, 8). Their quantification in biological fluids, such as blood, is also characterized by lack of invasiveness and relatively low cost (8).

An early and accurate diagnosis of subclinical infection can indeed improve bTB control strategies (7). Another important achievement would be the identification of biomarkers able to differentiate early infection from a late stage of the disease (6, 7). In this way, the available resources can be focused toward removing animals that pose transmission risks to preserve those with higher economic, and/or genetic value (7). Previous studies reported that bTB infection in cattle could be enhanced by a combination of IFN-γ and the related chemokine IFN-γ-induced protein 10 (IP-10) (7). Other cytokines are involved in immune response against this pathogen, and their potential use as biomarkers of *M. bovis* requires further investigation (7, 9).

In our work, we evaluated the ability of 16 key immune cytokines to identify *M. bovis* infected cattle. In addition, we investigated whether these cytokines were able to differentiate infected animals presenting or not TB-like lesions at post-mortem examinations, thereby exploring their value not only for diagnosis but also for the immunological staging of bTB.

## 2 Materials and methods

### 2.1 Ethical statements

Cattle used in this research were monitored within the context of the official TB eradication program, carried on in accordance with the Italian and European legislation (Regulation (EU) 2016/429, Regulation (EU) 2020/689, O.M. 28/05/2015 and subsequent amendments, DGRC 104/2022 and subsequent amendments) (10–13). No animal was harmed or killed for the specific purpose of this study, in compliance with the European Directive 210/63/UE and the Italian regulation D Lgs n° 26/2014.

### 2.2 Animals and Study Design

Fifty-five cattle were tested in this study.

Uninfected cattle were selected from Officially Tuberculosis-Free (OTF) herds in the Campania region (Italy). All the animals of this group had tested negative to the SIT or IFN-γ screening carried out over the last 6 years.

*M. bovis* infected and affected cattle belonged to herds with confirmed TB outbreaks. TB infection status was determined by ante-mortem tests: the single intradermal tuberculin test (SIT) and the IFN-γ test (see 2.3). Animals that tested positive were slaughtered in accordance with the current legislation, and then the presence of TB-like lesions was evaluated. The organs were sent to the laboratory of the IZS of Mezzogiorno to detect the presence of *M. bovis DNA* (See 2.4).

Animals were divided into three groups based on ante-mortem (SIT, IFN-γ test) and post-mortem (presence of TB-lesion and *M. bovis* DNA) tests: healthy (animals from OTF herds, IFN-γ test negative; N = 19), infected (IFN-γ test positive, no TB-like lesions at post-mortem examination, PCR negative; N = 17), and affected (IFN-γ test positive, visible TB-like lesions, PCR positive; N = 19), as previously described (14).

### 2.3 Whole blood stimulation and IFN-γ test

Blood samples were collected from the jugular vein using heparin as anticoagulant (lithium-heparin vacutainer tubes, BD Biosciences) and delivered to the laboratory of the IZS of Mezzogiorno (Portici) within 8 h from sampling.

For each animal, whole blood samples were dispensed in aliquots (1 ml) using 48 well plates. Samples were stimulated with 10 μg of PPD-B (BOVIGAM™ Thermo-Fisher Scientific, Schlieren, Switzerland). Phosphate-buffered saline (PBS) was used as Nil Control Antigen, whereas Pokeweed Mitogen (PWM, final concentration 1 μg/ml) was used as a control of lymphocyte reactivity. An additional aliquot was stimulated with 10 μg of avian PPD (PPD-A) (Thermo-Fisher Scientific). After an incubation step of 18–24 h at 37 °C, plasmas were collected to determine the amount of IFN-γ (within the context of the TB control program) and other key immune cytokines (see 2.5).

Levels of IFN-γ were quantified using the BOVIGAM™ sandwich ELISA test, following manufacturer’s instructions (Life Technologies, Thermo-Fisher Scientific). The absorbance of each well was read with a microplate reader (iMark™ BIORAD) using a 450 nm filter. Samples were regarded as positive for *M. bovis* when the differences between PPD-B - PBS and PPD-B – PPD-A were ≥ 0.1 OD, following the European Standard Operating Procedures (SOP/004/EURL) of the European Union Reference Laboratory for Bovine Tuberculosis (EURL-TB).

### 2.4 Post-mortem diagnostic tests

Animals tested positive to SIT or IFN-γ tests were slaughtered in compliance with the official TB eradication program. Post-mortem examinations were carried out to detect the presence of typical TB lesions: several tissue samples were collected and sent to the laboratory of the IZS del Mezzogiorno (Portici), as previously described (15). In these samples, PCR was also carried out to detect *Mycobacterium tuberculosis complex* (MTBC) DNA, following the WOAH Terrestrial Manual protocols (**Supplementary Table 1**) (16).

### 2.5 Measurement of cytokine levels in plasma after stimulations

Values of key immune cytokines in plasma of PBS, PPD-B, and PWM samples were evaluated using ELISAs. Twelve cytokines were determined using multiplex ELISA (IL-1α, IL-4, IL-6, IL-10, IL-17, IL-36Ra, MIP-1α, IP-10, MCP-1, TNF, VEGF-A, IFN-γ), using the Bovine Cytokine/Chemokine Magnetic Bead Panel Multiplex assay (Merck Millipore, Darmstadt, Germany) and a Bioplex MAGPIX Multiplex Reader (Bio-Rad, Hercules, CA, USA), as previously described (14). Four cytokines (IL-23, IL-27, IL-35, THBS-1) were measured using singleplex ELISA, following manufacturer’s instruction: Bovine Interleukin 23 ELISA Kit, Bovine Interleukin 27 ELISA Kit, Bovine Interleukin 35 ELISA Kit (all Mybiosource, San Diego, CA, USA), and ELISA Kit for Thrombospondin 1 (THBS-1) (Cloud-Clone Corp, Katy, TX, USA). For all the cytokines under study, samples were investigated in duplicate (two technical replicates).

The levels of *M. bovis* specific cytokine responses were determined by subtracting baseline values (PBS, nil control) from those measured in the PPD-B stimulated antigen condition.

### 2.6 Statistical analysis

Before ANOVA procedures, the normality of the traits was checked by computing skewness and kurtosis using PROC UNIVARIATE.

Levels of the 16 tested cytokines were analyzed using the general linear model (GLM) to estimate the mean response for each stimulus (PBS, PPD-B, and PWM) within the three animal groups (healthy, infected, and affected):


Yjk=μ+Gj+ejk

where Yjk is the trait measured for each animal, μ is the overall mean, Gj is the fixed effect of the stimuli (j = 3 levels: PBS, PPD-B, and PWM), and ejk is the random residual effect of each observation.

The statistical significances of all traits and least-square means were assessed by Dunnett’s multiple test in the GLM procedure.

Additionally, the difference (Δ_cytokine) between the level of each specific cytokine measured in the *M. bovis* antigen condition (PPD-B) and its baseline concentration (PBS) was analyzed by Tukey multiple comparison test and displayed by GraphPad Prism 10.01 (GraphPad Software Inc., La Jolla, CA, United States). The significance level for both statistical analyses was set at a p-value< 0.05.

Pearson correlation test was also performed on each Δ_cytokine.

A multivariate approach was conducted using canonical discriminant analysis (CDA) on all the tested 16 Δ_cytokines and on the 5 (IFN-γ, IL-4, IL-17, IP-10, and TNF) that showed significant differences among the three groups by the CANDISC Procedure.

The CDAs were conducted by categorizing animals into healthy, infected, and affected groups.

All statistical analyses were performed with SAS software version 9.4.

## 3 Results

Whole blood samples from healthy (N = 19), infected (N = 17), and affected (N = 19) cattle were stimulated with a specific *M. bovis* antigen (PPD-B), alongside the nil control antigen (PBS) and a control of lymphocyte reactivity (PWM). After 18–24 h, the release of 16 key immune cytokines was determined through singleplex and multiplex ELISA.

Statistically significantly higher levels of IFN-γ, IL-1α, IL-4, IL-10, IL-17, and IP-10 were detected in PWM-stimulated samples compared to PBS-samples (**Table 1**). This indicated that leukocyte reactivity was not altered by inadequate preservation of the sample or immunosuppression of the cattle due to other pathological events or treatments (e.g., corticosteroids).

**Table 1:** Production of cytokines in whole blood from healthy and *Mycobacteriumm bovis* infected and affected cattle.

	PBS	PPD-B	PWM	PBS-PPD-B	PBS-PWM
	LSM ± SE	LSM ± SE	LSM ± SE	p-value	p-value
Healthy					
Cytokines					
IFN-γ	2 ± 129	7 ± 129	970 ± 129	0.9995	**0.0001**
IL-1α	57 ± 24	86 ± 24	180 ± 24	0.6011	**0.0011**
IL-4	101 ± 81	104 ± 81	991 ± 81	0.9993	**0.0001**
IL-6	650 ± 194	2383 ± 194	704 ± 194	**0.0001**	0.9719
IL-10	247 ± 78	351 ± 78	1210 ± 78	0.5466	**0.0001**
IL-17	2 ± 30	5 ± 30	266 ± 30	0.9954	**0.0001**
MIP-1α	2925 ± 271	4246 ± 271	4979 ± 271	**0.0022**	**0.0001**
IL-36Ra	307 ± 37	318 ± 37	310 ± 37	0.9664	0.9971
IP-10	1446 ± 228	1673 ± 228	4022 ± 228	0.7056	**0.0001**
MCP-1	5425 ± 295	5431 ± 295	5316 ± 295	1.0000	0.9514
TNF	2513 ± 1217	3273 ± 1217	6035 ± 1217	0.8686	0.0826
VEGF-A	229 ± 20	192 ± 20	176 ± 20	0.3151	0.1122
IL-23	208 ± 28	211 ± 28	225 ± 28	0.9978	0.8973
IL-27	118 ± 35	91 ± 35	93 ± 33	0.8033	0.8303
IL-35	277 ± 27	270 ± 27	285 ± 27	0.9774	0.9705
THBS1	2236 ± 1645	2478 ± 1645	2661 ± 1865	0.9923	0.9792
Infected					
Cytokines					
IFN-γ	5 ± 84	413 ± 84	944 ± 84	**0.0024**	**0.0001**
IL-1α	44 ± 28	146 ± 28	193 ± 28	**0.0241**	**0.0008**
IL-4	69 ± 46	75 ± 46	262 ± 46	0.9929	**0.0084**
IL-6	1337 ± 309	3545 ± 309	1013 ± 309	**0.0001**	0.6801
IL-10	210 ± 121	373 ± 121	1024 ± 121	0.5361	**0.0001**
IL-17	2 ± 39	13 ± 39	357 ± 39	0.9687	**0.0001**
MIP-1α	2612 ± 745	4848 ± 745	5594 ± 745	0.0709	**0.0128**
IL-36Ra	459 ± 68	461 ± 68	484 ± 68	0.9996	0.9515
IP-10	1708 ± 289	3121 ± 289	3399 ± 289	**0.0024**	**0.0003**
MCP-1	5489 ± 559	5589 ± 559	5546 ± 559	0.9882	0.9961
TNF	1738 ± 492	3109 ± 492	4759 ± 492	0.0978	**0.0001**
VEGF-A	231 ± 21	142 ± 21	169 ± 21	**0.0101**	0.084
IL-23	250 ± 19	230 ± 19	227 ± 19	0.6775	0.608
IL-27	75 ± 37	78 ± 37	73 ± 37	0.9965	0.9993
IL-35	326 ± 44	347 ± 44	332 ± 44	0.9256	0.9935
THBS1	863 ± 258	729 ± 250	726 ± 250	0.9031	0.8995
Affected					
Cytokines					
IFN-γ	7 ± 170	825 ± 170	1298 ± 170	**0.0025**	**0.0001**
IL-1α	31 ± 23	124 ± 23	207 ± 23	**0.0141**	**0.0001**
IL-4	95 ± 46	109 ± 46	400 ± 46	0.9662	**0.0001**
IL-6	885 ± 414	3500 ± 414	675 ± 414	**0.0001**	0.9106
IL-10	124 ± 73	266 ± 73	847 ± 73	0.2881	**0.0001**
IL-17	2 ± 93	30 ± 93	520 ± 93	0.9671	**0.0005**
MIP-1α	3223 ± 389	4927 ± 389	4276 ± 389	**0.0061**	0.1094
IL-36Ra	331± 30	323± 30	349± 30	0.9776	0.883
IP-10	1612 ± 185	3179 ± 185	3093 ± 185	**0.0001**	**0.0001**
MCP-1	5953 ± 328	6076 ± 328	5758 ± 328	0.9497	0.8797
TNF	978 ± 414	3332 ± 414	4667 ± 414	**0.0004**	**0.0001**
VEGF-A	218 ± 19	150 ± 19	198 ± 19	**0.0246**	0.6611
IL-23	222 ± 20	223 ± 20	228 ± 20	0.9994	0.9722
IL-27	110 ± 43	111 ± 43	113 ± 43	0.9999	0.9991
IL-35	369 ± 41	379 ± 41	377 ± 41	0.9797	0.9871
THBS1	683 ± 253	593 ± 247	726 ± 247	0.9534	0.9891

P value< 0.05 were considered statistically significant and are marked in bold.

Whole blood was stimulated with PBS (nil control) or *M. bovis* antigen (PPD-B) or PWM (control of lymphocyte reactivity). Levels of sixteen cytokines were determined through multiplex and singleplex ELISA. LSM (Least Squares Mean) and SE (Standard Estimated Error) values and statistical differences between conditions (p-value) are presented.

Differences between PPD-B-stimulated samples and PBS-samples were also identified. In all groups, higher levels of IL-6 and MIP-1α, were observed in the PPD-B compared to the PBS conditions were observed for IL-6 and MIP-1α, suggesting an inflammatory response to the PPD-B antigen, regardless of previous animals’ exposure to *M. bovis* (**Table 1**). On the contrary, the infected and affected groups, but not healthy animals, presented statistically significantly higher levels of IFN-γ, IL-1α, and IP-10 in the PPD-B condition compared to the baseline control (PBS) (**Table 1**). Moreover, the infected and affected groups, but not healthy animals, presented lower levels of VEGF-A in the PPD-B condition compared to PBS, with statistical significance (p< 0.05). In the affected group only, we observed a higher release of TNF in response to stimulation with PPD-B compared to the PBS control, with statistical significance (p = 0.0004).

Then, *M. bovis* specific cytokine responses were analyzed. For each cytokine, the differences between the levels in the PPD-B and PBS conditions were quantified. These response differences were then compared among the three groups (healthy, infected, and affected).

**Figure 1:**
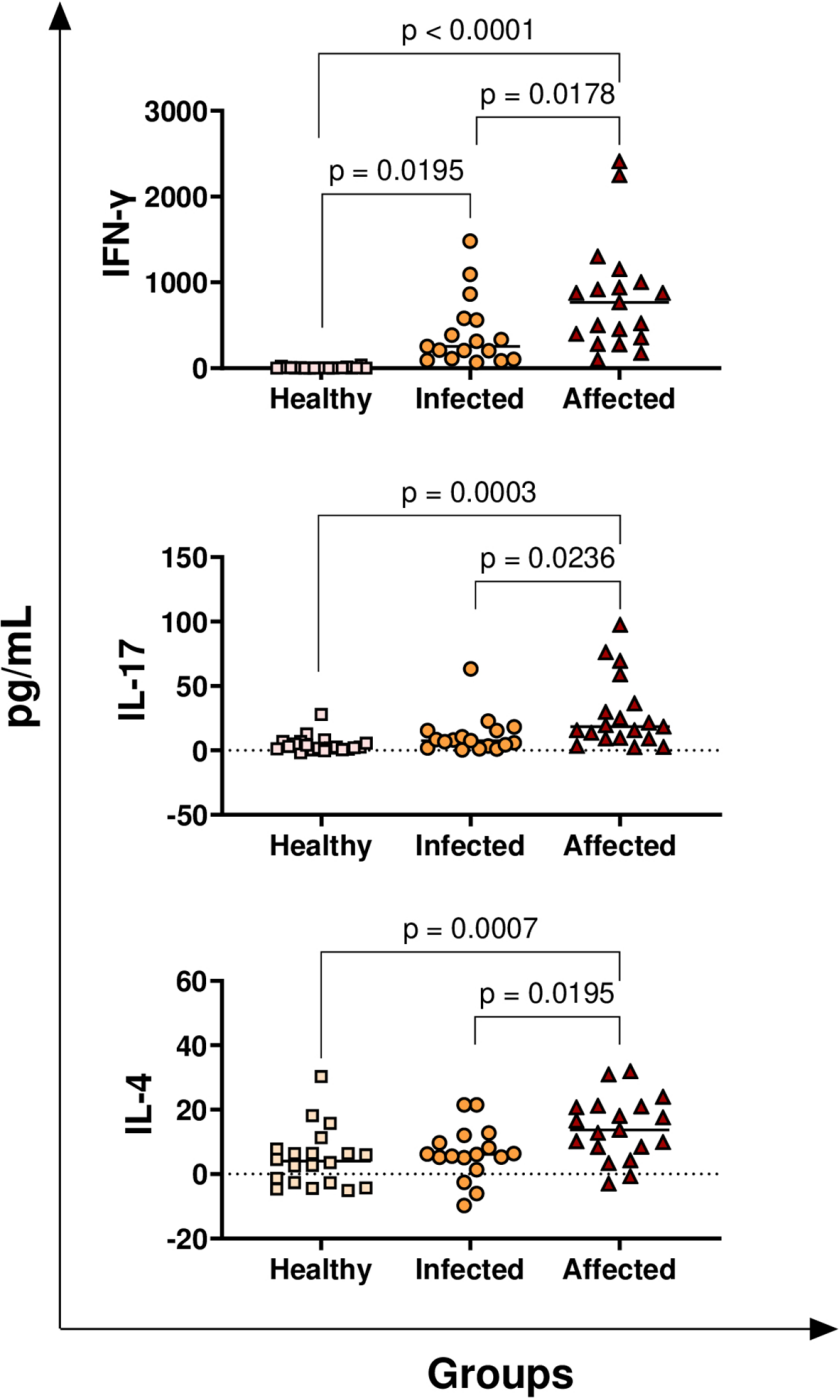
Release of *M. bovis* specific T-cell cytokines (IFN-γ, IL-4, IL-17) in healthy, infected, and affected cattle. Whole blood from healthy (N = 19), infected (N = 17), and affected (N = 19) cattle was collected using heparin as anticoagulant. Whole blood was stimulated with PPD-B, alongside PBS (nil control antigen). After 18–24 h, plasmas were collected, and levels of IFN-γ, IL-4, and IL-17 were quantified through multiplex ELISA. *M. bovis* specific cytokine responses were determined by subtracting PBS cytokine levels from those measured in the PPD-B condition. Differences between groups are displayed; p-values< 0.05 were considered statistically significant.

T-cell cytokines were first analysed: IFN-γ (hallmark of Th1 response), IL-4 (hallmark of Th2 response), and IL-17 (mainly released by Th17). Affected and infected animals released higher levels of *M. bovis*-specific IFN-γ (818 ± 99; 409 ± 105, respectively) compared to healthy cattle (5 ± 99) with p < 0.0001 and p< 0.0195, respectively (**Figure 1**). In addition, differences in *M. bovis*-specific IFN-γ levels were observed between the affected and infected groups (p = 0.0178) (**Figure 1**). Affected animals, but not infected cattle, released higher levels of *M. bovis* specific IL-4 and IL-17 compared to healthy cattle, with p < 0.0007 and p< 0.0003, respectively (**Figure 1**). In addition, affected animals released higher levels of *M. bovis* specific IL-4 and IL-17 compared to infected subjects, with p < 0.0195 and p< 0.0236, respectively (**Figure 1**).

**Figure 2:**
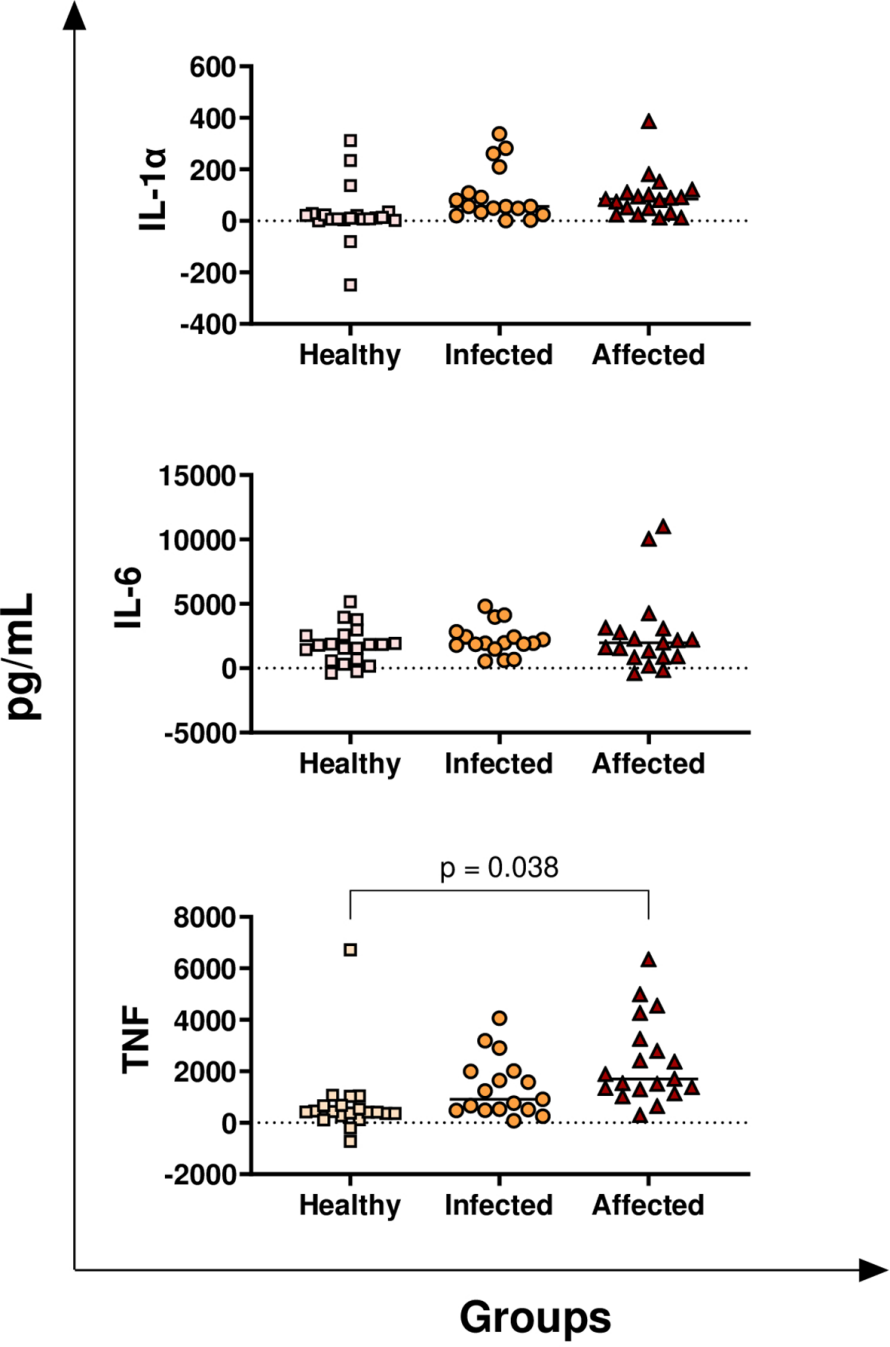
Release of *M. bovis* specific pro-inflammatory cytokines (IL-1α, IL-6, TNF) in healthy, infected, and affected cattle. Whole blood from healthy (N = 19), infected (N = 17), and affected (N = 19) cattle was collected using heparin as anticoagulant. Whole blood was stimulated with PPD-B, alongside PBS (nil control antigen). After 18–24 h, plasmas were collected, and levels of IL-1α, IL-6, and TNF, were quantified through multiplex ELISA. *M. bovis* specific cytokine responses were determined by subtracting PBS cytokines levels from those measured in the PPD-B condition. Differences between groups are displayed; p-values< 0.05 were considered statistically significant.

Release of *M. bovis* specific pro-inflammatory cytokines (IL-1α, IL-6, TNF) in healthy, infected, and affected cattle was then analysed. No significant differences were observed among groups for IL−1α or IL−6. In contrast, affected cattle showed higher levels of *M. bovis* specific TNF compared with healthy animals (**Figure 2**).

Subsequently, the release of *M. bovis* specific anti-inflammatory cytokines (IL-10, IL-36Ra) in the three groups was investigated. For both the anti-inflammatory IL-10 and the receptor antagonist IL-36Ra, no differences between groups were observed (**Supplementary Figure 1**).

**Figure 3:**
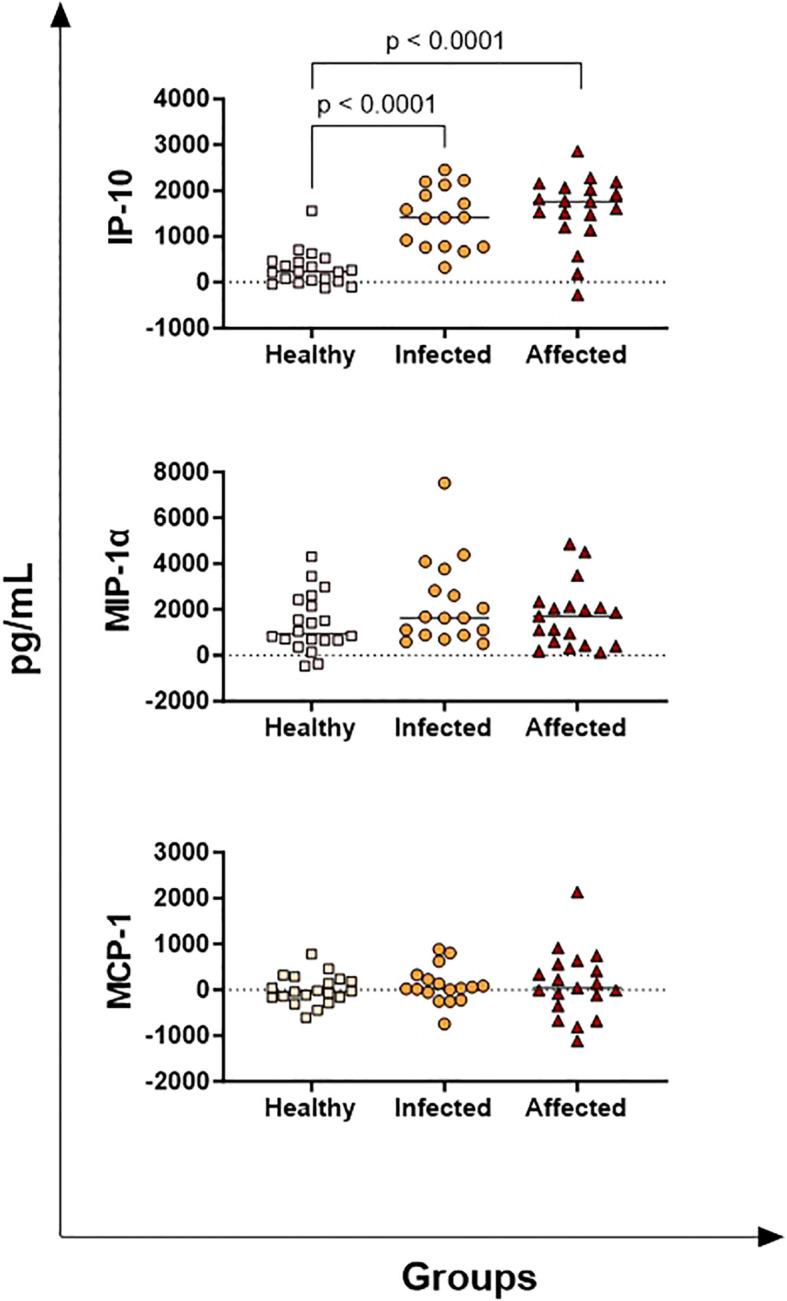
Release of *M. bovis* specific chemokines (IP-10, MIP-1α, MCP-1) in healthy, infected, and affected cattle. Whole blood from healthy (N = 19), infected (N = 17), and affected (N = 19) cattle was collected using heparin as anticoagulant. Whole blood was stimulated with PPD-B, alongside PBS (nil control antigen). After 18–24 h, plasmas were collected, and levels of IP-10, MIP-1α, and MCP-1 were quantified through multiplex ELISA. *M. bovis* specific cytokine responses were determined by subtracting PBS cytokine levels from those measured in the PPD-B condition. Differences between groups are displayed; p-values< 0.05 were considered statistically significant.

**Figure 4:**
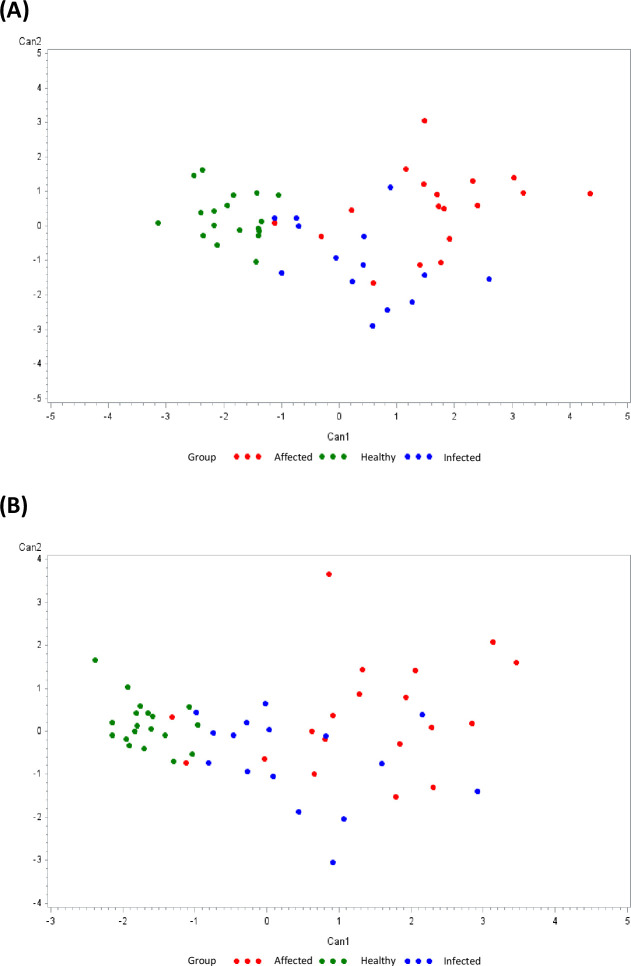
Plot from canonical discriminant analyses. In **(A)**, a multivariate approach was conducted using canonical discriminant analysis on 16 Δ_cytokines using SAS v9.4 (CANDISC Procedure). In **(B)**, a multivariate approach was conducted using canonical discriminant analysis on 5Δ_cytokines ((IFN-γ, IL-4, IL-17, IP-10, TNF) using SAS v9.4 (CANDISC Procedure). In both panels, animals belonging to the three groups (healthy (N = 19), infected (N = 17), affected (N = 19)) are displayed based on the two canonical functions (Can1, Can2).

IP-10, MIP-1α, and MCP-1 are chemokines that trigger leukocyte recruitment into the inflammatory site. We observed that affected and infected animals released higher levels of *M. bovis*-specific IP-10 (1567 ± 135; 1414 ± 148, respectively) compared to healthy cattle (227 ± 135), both with p< 0.0001 (**Figure 3**). No differences in *M. bovis*-specific IP-10 levels were instead observed between the affected and infected groups (**Figure 4**). For both MIP-1α and MCP-1, no differences between groups were detected (**Figure 3**).

IL-23, IL-27, and IL-35 are members of the IL-12 family, and their *M. bovis* specific levels in healthy, infected, and affected cattle were determined and compared. For all these cytokines, no differences among groups were observed (**Supplementary Figure 2**).

The levels of *M. bovis* specific VEGF-A (vascular endothelial growth factor) and THBS-1 (thrombospondin-1, an adhesive glycoprotein that mediates cell-to-cell and cell-to-matrix interactions) were also analyzed. Affected and infected animals released lower levels of *M. bovis*-specific VEGF-A (-68 ± 16; -88 ± 17, respectively) compared to healthy cattle (-37 ± 16), although without significance (p = 0.3769; p = 0.0816) (**Supplementary Figure 3**). For THBS-1, no statistically significant differences between healthy, infected, and affected cattle were observed (**Supplementary Figure 3**).

The correlation analysis didn’t highlight either high or significant interdependence among the tested variables, as shown in the **Supplementary Table 1**.

Finally, canonical discriminant analyses (CDA) were used to generate predictive cytokine profiles by groups, to identify potential diagnostic biomarkers of *M. bovis* infection. A CDA was performed with the 16 cytokines monitored in the study (**Figure 4A**). The first canonical (Can1) differentiates between healthy and *M. bovis* exposed animals, whereas the second canonical (Can2) could only modestly allow a separation between infected and affected cattle (**Figure 4A**). **Table 2A** reports the factor loading (FL) for each cytokine in canonical variables, but only Can 1 showed a positive and high correlation with Δ_IP-10 (FL = 0.82), Δ_IFN-γ (FL = 0.75), Δ_IL-17 (FL = 0.58), and Δ_IL-4 (FL = 0.52).

**Table 2:** Correlations between original variables and canonical functions for both canonical discriminant analyses.

A			B		
Δ_cytokine	Can 1	Can 2	Δ_cytokine	Can 1	Can 2
**Δ_IFN-γ**	**0.75**	0.15	**Δ_IFN-γ**	**0.76**	0.34
Δ_IL-1α	0.32	-0.11	**Δ_IL-4**	**0.55**	0.37
**Δ_IL-4**	**0.52**	0.37	**Δ_IL-17**	**0.58**	**0.54**
Δ_IL-6	0.22	-0.01	**Δ_IP-10**	**-0.89**	0.49
Δ_IL-10	0.17	-0.24	**Δ_TNF**	**0.50**	-0.35
**Δ_IL-17**	**0.58**	0.33			
**Δ_** MIP-1α	0.12	-0.26			
Δ_IL-36Ra	-0.15	-0.10			
**Δ_IP-10**	**0.82**	-0.30			
Δ_MCP1	0.04	-0.14			
Δ_TNF	0.49	0.21			
Δ_VEGF	-0.25	0.41			
Δ_IL-23	-0.08	0.29			
Δ_IL-27	0.27	-0.19			
Δ_IL-35	0.15	0.15			
Δ_THBS1	-0.23	0.15			

The heavier correlation coefficients are marked in bold.

The difference (Δ_cytokine) between the level of each specific cytokine measured in the antigen condition (PPD-B) and the corresponding baseline concentration (PBS), and their weight in the Can 1 and Can2, are presented: A Sixteen tested cytokines. B Five cytokines that significantly differentiate healthy and infected/affected animals (IFN-γ, IL-4, IL-17, IP-10, TNF).

To identify the most discriminating combination of cytokines, another CDA was performed using the 5 cytokines that presented statistically significant differences among groups (IP-10, IFN-γ, IL-17, IL-4, TNF). The resulting scatter plot of multivariate outcomes is shown in **Figure 4**. Using these 5 cytokines, Can1 could still differentiate healthy from *M. bovis* exposed animals, except for three animals (**Figure 4B**). The second canonical (Can2) could only modestly allow a separation between infected and affected cattle (**Figure 4B**). **Table 2B** reports the factor loading (FL) for each cytokine in canonical variables, showing a positive and high correlation with Δ_IP-10 (FL = 0.89), Δ_IFN-γ (FL = 0.76), Δ_IL-17 (FL = 0.58), and Δ_IL-4 (FL = 0.55) and Δ_TNF (FL = 0.50) in Can 1, while in Can 2 only **Δ_**IL-17 had a high FL (0.54).

## 4 Discussion

Bovine tuberculosis is a zoonotic disease threatening the cattle industry worldwide. To date, several studies have been conducted to improve its diagnosis and understanding, including the discovery of new biomarkers (7). *M. bovis* infection triggers the development of a cell-mediated immune response, which precedes humoral responses; accordingly, IFN-γ remains one of the most widely used biomarkers for detecting *M. bovis* infection in cattle and other species. Several additional cytokines are involved in immune response against this pathogen and their potential use as biomarkers of *M. bovis* infection in cattle requires further investigation (7, 9). In this study, we evaluated the utility of 16 key immune cytokines as diagnostic biomarkers for *M. bovis* infection in cattle.

IFN-γ is a cytokine mainly released by NK and activated T cells in the framework of a Th1 response (17). It is routinely used in the diagnosis of *M. bovis* infection in cattle, and in our work, it was indeed able to discriminate healthy from infected animals. Differences were also observed between cattle presenting (‘affected group’) or lacking (‘infected group’) visible TB-lesions, suggesting that the magnitude of the antigen-specific IFN-γ response might increase with disease progression and lesion development during *M. bovis* infection.

IL-4 is regarded as a signature cytokine of a Th2 response (18). Previous studies reported that this cytokine was able to distinguish *M. bovis* infected cattle from healthy subjects, although IL-4 response was delayed compared to the IFN-γ response (19). It was speculated that the IL-4 response reflected a switch from Th1- to Th2-dominated responses in the later stage of *M. bovis* infection (7). Accordingly, we observed that affected cattle released higher PPD-B-specific IL-4 compared to those belonging to the infected and control groups. Our data suggest that this cytokine might be useful for differentiating stages of bTB infection in cattle.

IL-17 is a cytokine mainly released by Th17 and γδ-T cells and it is a hallmark of a Th17 response (20). Previous studies reported that *M. bovis* infected cattle presented higher PPD-B-specific release of IL-17 compared to healthy subjects (21, 22). In addition, Blanco and collaborators observed that IL-17 expression was associated with the presence of TB-lesions in infected cattle (23). Accordingly, we observed that cattle, belonging to the affected group (with visible TB-lesions), released higher PPD-B-specific IL-17 compared to those belonging to the infected and control groups, suggesting that this cytokine might be useful in cattle to distinguish bTB infection stage.

IL-1α, IL-6, and TNF are key pro-inflammatory cytokines released during the early stages of infection, which enhance inflammation and trigger the release of chemokines, which in turn enhance the recruitment of leukocytes to the infection focus. We observed no differences among groups in terms of PPD-B specific release of IL-1α and IL-6, whereas affected cattle presented higher levels of TNF compared to healthy subjects. Previous studies in humans described that antigen-specific release of TNF by CD4^+^ T cells was able to distinguish patients with active and latent infection (24) and we recently observed that the frequency of TNF^+^ producing CD4^+^ T cells enabled discrimination between infected/exposed and non-infected Mediterranean buffaloes (25). In agreement, we previously reported that Mediterranean buffaloes with visible TB lesions at the slaughterhouse released higher levels of TNF in response to PPD-B stimulation compared to healthy subjects and those reactive only to the IFN-γ assay (14). Overall, these data suggest that TNF might be a valuable biomarker for distinguishing bTB infection stages across different host species, including cattle.

Two anti-inflammatory cytokines were then evaluated: IL-10 and IL-36Ra (26, 27). Previous studies reported that IL-10 promotes *M. bovis* survival within macrophages (28) and plays an important role in granuloma formation (29). Nevertheless, no differences between groups were observed in terms of PPD-B specific IL-10 release and this might be due to the diverse cytokine pattern in cells in peripheral blood (evaluated in our study) compared with those present within granulomatous lesions, where IL-10 production is typically more pronounced. No differences were also observed for IL-36Ra in all tested cattle, according to what we previously observed in Mediterranean buffaloes (14).

Chemokines are low-molecular-weight mediators that promote cell recruitment to infected tissues (30–32). We observed no differences between groups in terms of PPD-B specific release of MIP-1α and MCP-1, whereas both infected and affected groups presented higher levels of IP-10 compared to healthy subjects. In agreement, several studies in cattle and buffaloes showed the utility of this chemokine in identifying *M. bovis* infected animals (3, 14, 33, 34). Studies in humans reported indeed that IP-10 can be a biomarker for tuberculosis in both adults and children. However, IP−10 does not reliably distinguish active TB from latent TB (35, 36). In agreement, our data suggest that IP-10 is not informative for differentiating stages of bTB in cattle, but it might improve early detection of the infection. Future studies with a larger number of animals will be essential to confirm these findings and to assess whether IP-10 can improve early diagnosis of *M. bovis*.

IL-23, IL-27, and IL-35 are members of the IL-12 family (37) and little is known about their role during *M. bovis* infection. We observed no differences among healthy, infected, and affected cattle. Our data disagree with those of Sharma and co-authors, where researchers described that PBMC from *M. bovis* infected cattle presented higher expression of IL-23 in response to PPD-B stimulation compared to those of healthy controls (38). This difference might be due to post-transcriptional mechanisms that inhibit IL-23 protein release from PBMC, despite elevated mRNA expression, in response to PPD-B stimulation.

VEGF-A stimulates angiogenesis and plays a key role in the maintenance of the vascular and lymphatic systems (39) and its role during *M. bovis* infection in cattle is largely unexplored. Previous studies in humans and mice reported that VEGF-A is implicated in granuloma formation and likely enhances tuberculosis spread in the host (40, 41). Higher serum levels of this growth factor were observed in human patients with symptoms of tuberculosis compared to healthy controls (40). Surprisingly, in our study we observed that cattle from both the infected and the affected groups, but not healthy controls, released lower levels of VEGF-A in response to PPD-B stimulation compared to nil control (PBS). Similar results were previously reported in Mediterranean Buffaloes (14). We might speculate that this downregulation is due to higher PPD-B-specific release of IFN-γ in the infected/affected groups compared to controls. Supporting this hypothesis, previous studies reported that IFN-γ can suppress VEGF-A release by monocytes (42).

THBS-1 is an adhesive glycoprotein which mediates cell-to-cell and cell-to-matrix interactions (43). In cattle, *THBS-1* was reported to be down-regulated in cattle experimentally infected with *M. bovis* (44) and another study described that healthy and naturally infected bTB cattle presented a differential transcription of the *THBS-1* gene (45). On the contrary, in our study, we observed no differences between healthy and *M. bovis* exposed cattle; this might be due to post-transcriptional mechanisms which nullify the difference between groups.

Finally, we aimed to identify the cytokine combinations that better differentiated the three groups. Two canonical analyses were performed: the first one included all the sixteen cytokines analyzed in the study, and the second one included only the five cytokines (IFN-γ, IP-10, IL-4, IL-10, TNF) with statistical differences among groups. In both analyses, a clear differentiation was observed between healthy and *M. bovis* exposed animals, whereas the separation between infected and affected cattle remained modest. Our data suggest that the quantitative determination of IFN-γ and the related chemokine IP-10 could be useful in the diagnosis of *M. bovis* infection in cattle, in agreement with previous studies (7). These preliminary observations should be validated to establish whether the parallel measurement of these two cytokines can improve the diagnosis of *M. bovis* infection in cattle in terms of sensitivity and specificity, and whether IP-10 could allow an earlier identification of infected cattle. In addition, our data suggested that IL-4, IL-10, and TNF might be useful to distinguish cattle in diverse stages of TB infection. Future studies on a larger set of samples will be essential to establish whether the combined measurement of these three cytokines can allow the identification of cattle with limited transmission risks, which might allow temporary retention of animals with high economic and/or genetic value under appropriate management and biosecurity conditions.

In parallel to the discovery of new biomarkers, another main area of bTB research is the identification of new antigens to improve IGRA specificity. PPD-B provides a wide variety of antigens that can be presented to lymphocytes, reflecting the range of antigens to which the host is exposed during infection, but shares epitopes with other mycobacteria, thus it shows limited specificity (46). Several studies investigated new antigens to improve IGRA specificity, such as ESAT6 and CFP10, which are potent T-cell–stimulating proteins secreted by *M. tuberculosis* and M*. bovis* (47–49). More recently, more complex antigen formulations have been evaluated in both IGRA and IDT, such as DST-F (ESAT-6/CFP-10 plus Rv3615c) and MDT, which comprises ESAT-6/CFP-10, Rv3615c, Rv3020c, Rv1789, Rv3478, and Rv3810 (50, 51). Other studies investigated the ability of the protein complex P22 to improve bTB diagnosis (52, 53). P22 is composed of several immunodominant antigens recognized by T cells, including MPB70, MPB83, ESAT-6, and CFP-10, and may provide greater specificity than PPD-B (53).

Future studies should combine both strategies (discovery for new biomarkers and identification of more specific antigens) to improve bTB diagnosis. The selected cytokines (IFN-γ, IP-10, IL-4, IL-10, TNF) should be quantified *in vitro* not only in samples stimulated with PPD-B, but also in response to stimulation with other antigens such as ESAT-6, ESAT10, or P22.

Overall, the data generated in our study provide a foundation to improve both the diagnosis and the immunological staging of *M. bovis* infection in cattle.

## Data Availability

The raw data supporting the conclusions of this article will be made available by the authors, without undue reservation.

## Supplementary material

Supplementary Figure 1 Release of *M. bovis* specific anti-inflammatory cytokines (IL-10, IL-36Ra) in healthy, infected, and affected cattle. Whole blood from healthy (N = 19), infected (N = 17), and affected (N = 19) cattle was collected using heparin as anticoagulant. Whole blood was stimulated with PPD-B, alongside PBS (nil control antigen). After 18–24 h, plasmas were collected, and levels of IL-10 and IL-36Ra were quantified through multiplex ELISA. *M. bovis* specific cytokine responses were determined by subtracting PBS cytokine levels from those measured in the PPD-B condition. Differences between groups are displayed; p-values< 0.05 were considered statistically significant.

Supplementary Figure 2 Release of *M. bovis* specific cytokines of the IL-12 family (IL-23, IL-27, IL-35) in healthy, infected, and affected cattle. Whole blood from healthy (N = 19), infected (N = 17), and affected (N = 19) cattle was collected using heparin as anticoagulant. Whole blood was stimulated with PPD-B, alongside PBS (nil control antigen). After 18–24 h, plasmas were collected, and levels of IL-23, IL-27, and IL-35 were quantified through singleplex ELISAs. *M. bovis* specific cytokine responses were determined by subtracting PBS cytokine levels from those measured in the PPD-B condition. Differences between groups are displayed; p-values< 0.05 were considered statistically significant.

Supplementary Figure 3 Release of other *M. bovis* specific cytokines (VEGF-A, THMS-1) in healthy, infected, and affected cattle. Whole blood from healthy (N = 19), infected (N = 17), and affected (N = 19) cattle was collected using heparin as anticoagulant. Whole blood was stimulated with PPD-B, alongside PBS (nil control antigen). After 18–24 h, plasmas were collected, and levels of VEGF-A, and THBS-1 were quantified through singleplex and multiplex ELISAs, respectively. *M. bovis* specific cytokine responses were determined by subtracting PBS cytokines levels from those measured in the PPD-B condition. Differences between groups are displayed; p-values< 0.05 were considered statistically significant.

## References

[1] Cousins DV . Mycobacterium bovis infection and control in domestic livestock. Rev Sci Tech (International Office Epizootics). (2001) 20:71–85. doi: 10.20506/rst.20.1.12632 11288521

[2] Sawyer J Rhodes S Jones GJ Hogarth PJ Vordermeier HM . Mycobacterium bovis and its impact on human and animal tuberculosis. J Med Microbiol. (2023) 72:1769. doi: 10.1099/jmm.0.001769, PMID: 37962183

[3] Coad M Doyle M Steinbach S Gormley E Vordermeier M Jones G . Simultaneous measurement of antigen-induced CXCL10 and IFN-γ enhances test sensitivity for bovine TB detection in cattle. Vet Microbiol. (2019) 230:1–6. doi: 10.1016/j.vetmic.2019.01.007, PMID: 30827373

[4] Srinivasan S Jones G Veerasami M Steinbach S Holder T Zewude A . A defined antigen skin test for the diagnosis of bovine tuberculosis. Sci Adv. (2019) 5:eaax4899. doi: 10.1126/sciadv.aax4899, PMID: 31328169 PMC6636981

[5] Schiller I Oesch B Vordermeier HM Palmer MV Harris BN Orloski KA . Bovine tuberculosis: A review of current and emerging diagnostic techniques in view of their relevance for disease control and eradication. Transboundary Emerging Dis. (2010) 57:205–20. doi: 10.1111/j.1865-1682.2010.01148.x, PMID: 20561288

[6] Willgert K Cliff M Meinke S Messina D Broom DM Wood J . Burden of bovine tuberculosis on animal health, welfare and production: A systematic review. Transbound Emerg Dis. (2025) 2025:6541298. doi: 10.1155/tbed/6541298, PMID: 41098524 PMC12520801

[7] Smith K Kleynhans L Warren RM Goosen WJ Miller MA . Cell-mediated immunological biomarkers and their diagnostic application in livestock and wildlife infected with Mycobacterium bovis. Front Immunol. (2021) 12:639605. doi: 10.3389/fimmu.2021.639605, PMID: 33746980 PMC7969648

[8] Liu C Chu D Kalantar-Zadeh K George J Young HA Liu G . Cytokines: from clinical significance to quantification. Adv Sci. (2021) 8:e2004433. doi: 10.1002/advs.202004433, PMID: 34114369 PMC8336501

[9] Palmer MV Thacker TC Rabideau MM Jones GJ Kanipe C Vordermeier HM . Biomarkers of cell-mediated immunity to bovine tuberculosis. Veterinary Immunol Immunopathol. (2020) 220:109988. doi: 10.1016/j.vetimm.2019.109988, PMID: 31846797

[10] Regulation (EU) 2016/429 of the European Parliament and of the council of 9 march 2016, on transmissible animal diseases and amending and repealing certain acts in the area of animal health (animal health law). Off J Eur Union. (2016) 84:1–208.

[11] Commission delegated regulation (EU) 2020/689 of 17 December 2019 supplementing regulation (EU) 2016/429 of the European Parliament and of the council as regards rules for surveillance, eradication programmes, and disease-free status for certain listed and emerging diseases. OJ L. (2020) 174:211–340.

[12] Italian Ministry of Health order 28 May 2015. Extraordinary veterinary police measures on tuberculosis, bovine and buffalo brucellosis, bovine and caprine brucellosis, enzootic bovine leukosis Vol. 144. Gazzetta Ufficiale Della Repubblica Italiana Serie Generale.

[13] . Italian Ministry of Health. Legislative decree No. 134 of August 5, 2022. Dispositions on the system of identification and registration of operators, stabiliations and animals for the adaptation of national legislation to the provisions of regulation (EU) 2016/429, pursuant to article 14, paragraph 2(a), (b), (g), (h), (i) and (p), of law April 22, 2021, no. 53.

[14] Franzoni G Signorelli F Mazzone P Donniacuo A De Matteis G Grandoni F . Cytokines as potential biomarkers for the diagnosis of Mycobacterium bovis infection in Mediterranean buffaloes (Bubalus bubalis). Front Vet Sci. (2024) 11:1512571. doi: 10.3389/fvets.2024.1512571, PMID: 39776597 PMC11703857

[15] Martucciello A Vitale N Mazzone P Dondo A Archetti I Chiavacci L . Field evaluation of the interferon gamma assay for diagnosis of tuberculosis in Mediterranean Buffalo (Bubalus bubalis) comparing four interpretative criteria. Front Vet Sci. (2020) 7:563792. doi: 10.3389/fvets.2020.563792, PMID: 33335916 PMC7736034

[16] World Organisation for Animal Health (WOAH) . Terrestrial Manual 2022 Chapter 3.1.13. Mammalian tuberculosis (infection with Mycobacterium tuberculosis complex). Available online at: https://www.woah.org/fileadmin/Home/eng/Health_standards/tahm/3.01.13_Mammalian_tuberculosis.pdf (Accessed September 25, 2025).

[17] Schroder K Hertzog PJ Ravasi T Hume DA . Interferon-γ: an overview of signals, mechanisms and functions. J Leukoc Biol. (2004) 75:163–89. doi: 10.1189/jlb.0603252, PMID: 14525967

[18] Gadani SP Cronk C Norris GT Kipnis J . Interleukin-4: A cytokine to remember. J Immunol. (2012) 189:4213–9. doi: 10.4049/jimmunol.1202246, PMID: 23087426 PMC3481177

[19] Thacker TC Palmer MV Waters WR . Associations between cytokine gene expression and pathology in Mycobacterium bovis infected cattle. Vet Immunol Immunopathol. (2007) 119:204–13. doi: 10.1016/j.vetimm.2007.05.009, PMID: 17628695

[20] Iwakura Y Ishigame H . The IL-23/IL-17 axis in inflammation. J Clin Invest. (2006) 116:1218–22. doi: 10.1172/JCI28508, PMID: 16670765 PMC1451213

[21] Walters WR Maggioli MF Palmer MV Thacker TC McGill JL Vordermeier HM . Interleukin-17A as a biomarker for bovine tuberculosis. Clin Vaccine Immunol. (2016) 23:168–80. doi: 10.1128/CVI.00637-15, PMID: 26677202 PMC4744917

[22] Steinbach S Vordemeier HM Jones J . GCD4+ and ψT cells are the main producers of IL-22 and IL-17A in lymphocytes from M. bovis infected cattle. Sci Rep. (2016) 6:29990. doi: 10.1038/srep29990, PMID: 27427303 PMC4947955

[23] Blanco FC Bianco MV Meikle V Garbaccio S Vagnoni L Forrellad M . Increased IL-17 expression is associated with pathology in a bovine model of tuberculosis. Tuberculosis (Edinb). (2011) 91:57–63. doi: 10.1016/j.tube.2010.11.007, PMID: 21185783

[24] Harari A Rozot V Bellutti Enders F Perreau M Stalder JM Nicod LP . Dominant TNF-α+ Mycobacterium tuberculosis–specific CD4+ T cell responses discriminate between latent infection and active disease. Nat Med. (2011) 17:372–6. doi: 10.1038/nm.2299, PMID: 21336285 PMC6570988

[25] Flores-Villalva S De Matteis G Grandoni F Scatà MC Donniacuo A Schiavo L . Polyfunctionality of CD4+ T lymphocytes in buffaloes and cattle: comparative antigen-specific cytokine responses in bovine tuberculosis infection. Front Immunol. (2025) 16:1608065. doi: 10.3389/fimmu.2025.1608065, PMID: 40990013 PMC12450747

[26] Mosser DM Zhang X . Interleukin-10: new perspectives on an old cytokine. Immunol Rev. (2008) 226:205–18. doi: 10.1111/j.1600-065X.2008.00706.x, PMID: 19161426 PMC2724982

[27] Garlanda C Dinarello CA Mantovani A . The interleukin-1 family: back to the future. Immunity. (2013) 39:1003–18. doi: 10.1016/j.immuni.2013.11.010, PMID: 24332029 PMC3933951

[28] Jensen K Stevens JM Glass EJ . Interleukin 10 knock-down in bovine monocyte-derived macrophages has distinct effects during infection with two divergent strains of Mycobacterium bovis. PloS One. (2019) 14:e0222437. doi: 10.1371/journal.pone.0222437, PMID: 31527895 PMC6748433

[29] Canal AM Pezzone N Cataldi A Zumarraga M Larzabal M Garbaccio S . Immunohistochemical detection of pro-inflammatory and anti-inflammatory cytokines in granulomas in cattle with natural Mycobacterium bovis infection. Res Vet Sci. (2017) 110:34–9. doi: 10.1016/j.rvsc.2016.10.006, PMID: 28159234

[30] Comerford I McColl SR . Mini-review series: focus on chemokines. Immunol Cell Biol. (2011) 89:183–4. doi: 10.1038/icb.2010.164, PMID: 21326315

[31] Menten P Wuyts A Van Damme J . Macrophage inflammatory protein-1. Cytokine Growth Factor Rev. (2002) 13:455–81. doi: 10.1016/s1359-6101(02)00045-x, PMID: 12401480

[32] Antonelli A Ferrari SM Giuggioli D Ferrannini E Ferri C Fallahi P . Chemokine (C-X-C motif) ligand (CXCL)10 in autoimmune diseases. Autoimmun Rev. (2014) 13:272–80. doi: 10.1016/j.autrev.2013.10.010, PMID: 24189283

[33] Bernitz N Kerr TJ Goosen WJ Clarke C Higgitt R Roos EO . Parallel measurement of IFN-γ and IP-10 in QuantiFERON^®^-TB Gold (QFT) plasma improves the detection of Mycobacterium bovis infection in African buffaloes (Syncerus caffer). Prev Vet Med. (2019) 169:104700. doi: 10.1016/j.prevetmed.2019.104700, PMID: 31311648

[34] Goosen WJ Parsons SDC Miller MA van Helden PD Warren RM Cooper D . The evaluation of candidate biomarkers of cell-mediated immunity for the diagnosis of Mycobacterium bovis infection in African buffaloes (Syncerus caffer). Vet Immunol Immunopathol. (2014) 162:198–202. doi: 10.1016/j.vetimm.2014.10.008, PMID: 25464825

[35] Whittaker E Gordon A Kampmann B . Is IP-10 a better biomarker for active and latent tuberculosis in children than IFN? PloS One. (2008) 3:e3901. doi: 10.1371/journal.pone.0003901, PMID: 19065267 PMC2588495

[36] Ruhwald M Dominguez J Latorre I Losi M Richeldi L Pasticci MB . A multicentre evaluation of the accuracy and performance of IP-10 for the diagnosis of infection with M. tuberculosis. Tuberculosis. (2011) 91:260–7. doi: 10.1016/j.tube.2011.01.001, PMID: 21459676

[37] Vignali DA Kuchroo VK . IL-12 family cytokines: Immunological playmakers. Nat Immunol. (2014) 13:722–8. doi: 10.1038/ni.2366, PMID: 22814351 PMC4158817

[38] Sharma S Filia G Leishangthem GD Sethi RS Kaur G . Studies on the immunological biomarkers of bovine tuberculosis in naturally infected cattle. Braz J Microbiol. (2026) 57:1. doi: 10.1007/s42770-025-01842-3, PMID: 41364133 PMC12690025

[39] Maniscalco WM D’Angio CT . Vascular endothelial growth factor. In: GJ Laurent and SD Shapiro, editors. Encyclopedia of respiratory medicine. 1st ed. Cambridge, MA, USA: Academic Press (2006) p. 413–8. doi: 10.1016/B0-12-370879-6/00434-8

[40] Saghazadeh A Rezaei N . Vascular endothelial growth factor levels in tuberculosis: A systematic review and meta-analysis. PloS One. (2022) 17:e0268543. doi: 10.1371/journal.pone.0268543, PMID: 35613134 PMC9132289

[41] Harding JS Herbath M Chen Y Rayasam A Ritter A Csoka B . VEGF-A from granuloma macrophages regulates granulomatous inflammation by a non-angiogenic pathway during mycobacterial infection. Cell Rep. (2019) 27:2119–2131.e6. doi: 10.1016/j.celrep.2019.04.072, PMID: 31091450

[42] Ray PS Fox PL . A post-transcriptional pathway represses monocyte VEGF-A expression and angiogenic activity. EMBO J. (2007) 26:3360–72. doi: 10.1038/sj.emboj.7601774, PMID: 17611605 PMC1933405

[43] Kvansakul M Adams JC Hohenester E . Structure of a thrombospondin C-terminal fragment reveals a novel calcium core in the type 3 repeats. EMBO J. (2004) 23:1223–33. doi: 10.1038/sj.emboj.7600166, PMID: 15014436 PMC381422

[44] Blanco FC Soria M Bianco MV Bigi F . Transcriptional response of peripheral blood mononuclear cells from cattle infected with Mycobacterium bovis. PloS One. (2012) 7:e41066. doi: 10.1371/journal.pone.0041066, PMID: 22815916 PMC3397951

[45] Klepp LI Eirin ME Garbaccio S Soria M Bigi F Blanco FC . Identification of bovine tuberculosis biomarkers to detect tuberculin skin test and IFNγ release assay false negative cattle. Res Vet Sci. (2019) 122:7–14. doi: 10.1016/j.rvsc.2018.10.016, PMID: 30447501

[46] Palmer MV Waters WR . Advances in bovine tuberculosis diagnosis and pathogenesis: what policy makers need to know. Vet Microbiol. (2006) 112:181–90. doi: 10.1016/j.vetmic.2005.11.028, PMID: 16326040

[47] Encinas M Marfil MJ Garbaccio S Barandiaran S Huertas P Morsella C . Mycobacterium bovis ESAT-6, CFP-10 and EspC antigens show high conservation among field isolates. Tuberculosis. (2018) 111:143–6. doi: 10.1016/j.tube.2018.06.007, PMID: 30029900

[48] van Pinxteren LAH Ravn P Agger EM Pollock J Andersen P . Diagnosis of tuberculosis based on the two specific antigens ESAT-6 and CFP10. Clin Diagn Lab Immunol. (2000) 7:155–60. doi: 10.1128/CDLI.7.2.155-160.2000, PMID: 10702486 PMC95842

[49] Aagaard C Govaerts M Meikle V Vallecillo AJ Gutierrez-Pabello JA Suarez-Güemes F . Optimizing antigen cocktails for detection of Mycobacterium bovis in herds with different Prevalences of bovine tuberculosis: ESAT6-CFP10 mixture shows optimal sensitivity and specificity. J Clin Microbiol. (2006) 44:4326–35. doi: 10.1128/JCM.01184-06, PMID: 17005738 PMC1698389

[50] Middleton S Steinbach S Coad M McGill K Brady C Duignan A . A molecularly defined skin test reagent for the diagnosis of bovine tuberculosis compatible with vaccination against Johne’s Disease. Sci Rep. (2021) 11:2929. doi: 10.1038/s41598-021-82434-7, PMID: 33536465 PMC7859399

[51] Middleton S Singh M Coad M Palmer S Holder T Steinbach S . 2025. Optimization of a molecularly defined tuberculin formulation: recombinant fusion proteins and epitope surgery. J Clin Microbiol. (2025) 63:e00552–25. doi: 10.1128/jcm.00552-25, PMID: 40879485 PMC12505890

[52] Giovannozzi S Martucciello A Rodríguez MD Iruela IM Boifava M Schiavo L . Evaluation of serological assays for intra vitam diagnosis of bovine tuberculosis in water buffalo (Bubalus bubalis). Front Microbiol. (2025) 16:1684425. doi: 10.3389/fmicb.2025.1684425, PMID: 41311486 PMC12646987

[53] Infantes-Lorenzo JA Moreno I Risalde MLÁ. Roy Á Villar M Romero B . Proteomic characterisation of bovine and avian purified protein derivatives and identification of specific antigens for serodiagnosis of bovine tuberculosis. Clin Proteom. (2017) 14:36. doi: 10.1186/s12014-017-9171-z, PMID: 29142508 PMC5669029

