# Supplementary figures and images for: Exploring alternative cytokines as potential biomarkers for Mycobacterium bovis infection in cattle
Source: Front Immunol. 2026 Feb 25;17:1786944. doi: 10.3389/fimmu.2026.1786944 (PMC12976014; doi:10.3389/fimmu.2026.1786944)

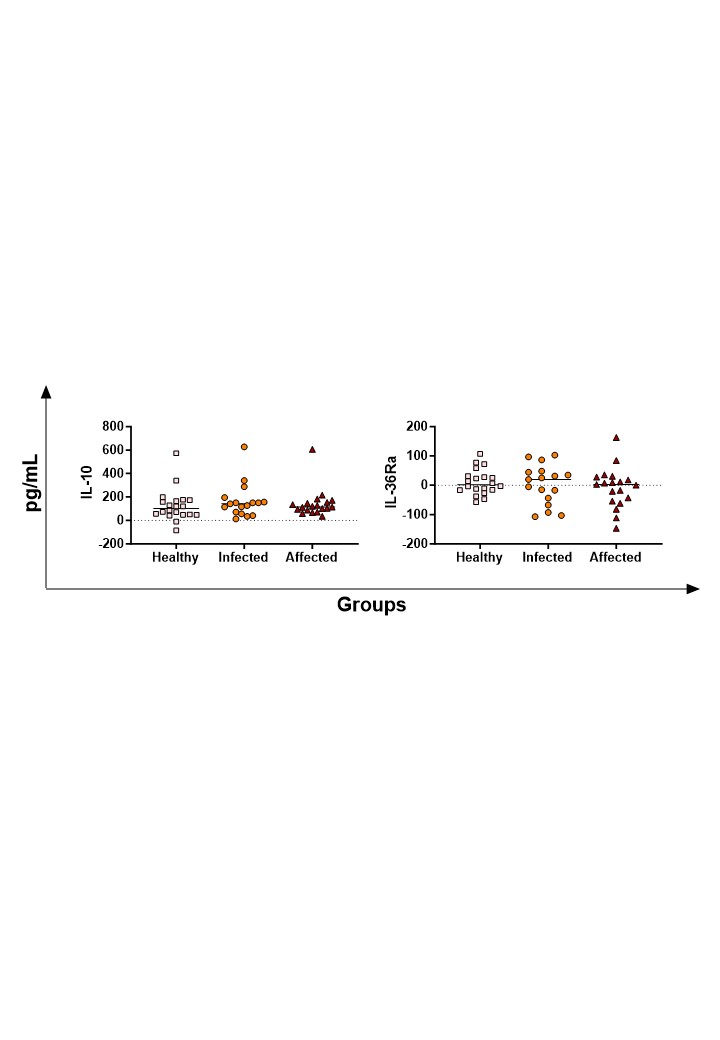

Supplement: Supplementary Figure 1 — Release of M. bovis specific anti-inflammatory cytokines (IL-10, IL-36Ra) in healthy, infected, and affected cattle. Whole blood from healthy (N = 19), infected (N = 17), and affected (N = 19) cattle was collected using heparin as anticoagulant. Whole blood was stimulated with PPD-B, alongside PBS (nil control antigen). After 18–24 h, plasmas were collected, and levels of IL-10 and IL-36Ra were quantified through multiplex ELISA. M. bovis specific cytokine responses were determined by subtracting PBS cytokine levels from those measured in the PPD-B condition. Differences between groups are displayed; p-values< 0.05 were considered statistically significant. [file Image1.jpeg]

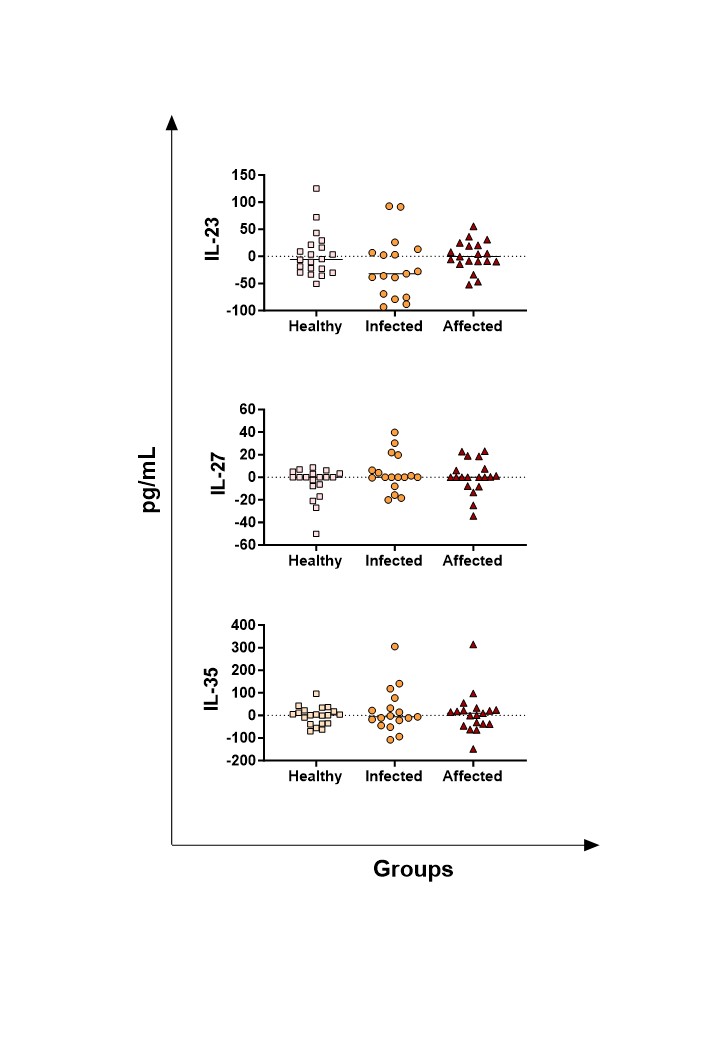

Supplement: Supplementary Figure 2 — Release of M. bovis specific cytokines of the IL-12 family (IL-23, IL-27, IL-35) in healthy, infected, and affected cattle. Whole blood from healthy (N = 19), infected (N = 17), and affected (N = 19) cattle was collected using heparin as anticoagulant. Whole blood was stimulated with PPD-B, alongside PBS (nil control antigen). After 18–24 h, plasmas were collected, and levels of IL-23, IL-27, and IL-35 were quantified through singleplex ELISAs. M. bovis specific cytokine responses were determined by subtracting PBS cytokine levels from those measured in the PPD-B condition. Differences between groups are displayed; p-values< 0.05 were considered statistically significant. [file Image2.jpeg]

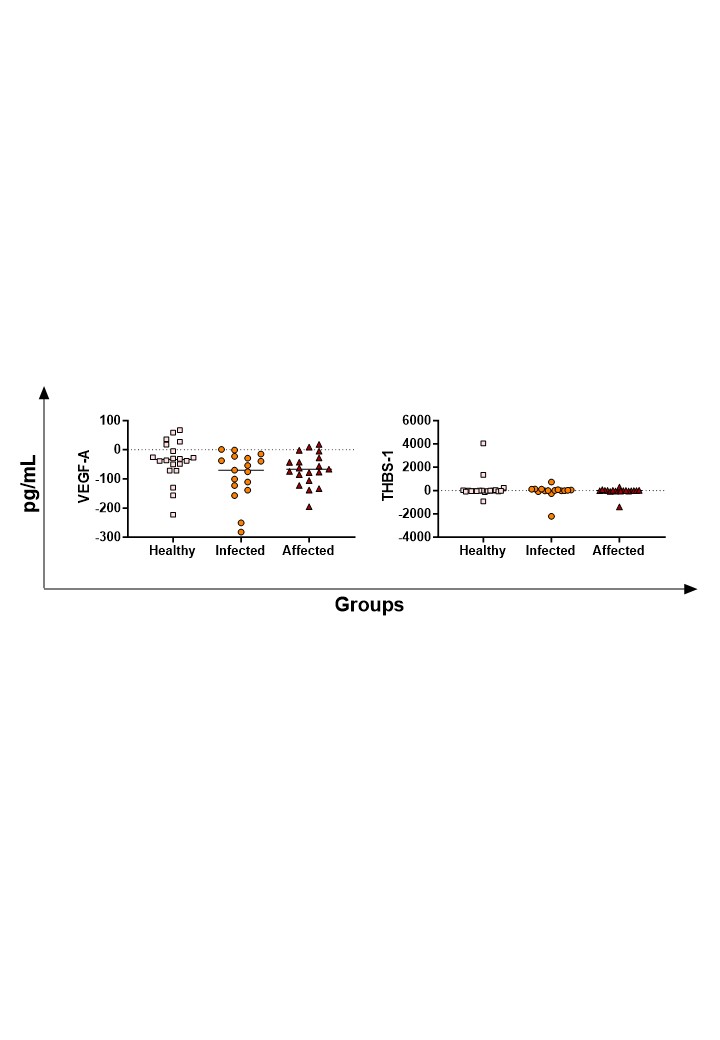

Supplement: Supplementary Figure 3 — Release of other M. bovis specific cytokines (VEGF-A, THMS-1) in healthy, infected, and affected cattle. Whole blood from healthy (N = 19), infected (N = 17), and affected (N = 19) cattle was collected using heparin as anticoagulant. Whole blood was stimulated with PPD-B, alongside PBS (nil control antigen). After 18–24 h, plasmas were collected, and levels of VEGF-A, and THBS-1 were quantified through singleplex and multiplex ELISAs, respectively. M. bovis specific cytokine responses were determined by subtracting PBS cytokines levels from those measured in the PPD-B condition. Differences between groups are displayed; p-values< 0.05 were considered statistically significant. [file Image3.jpeg]
