# Supplementary material for: Exploring alternative cytokines as potential biomarkers for Mycobacterium bovis infection in cattle
Source: Front Immunol. 2026 Feb 25;17:1786944. doi: 10.3389/fimmu.2026.1786944 (PMC12976014; doi:10.3389/fimmu.2026.1786944)
Supplement: Supplementary file 4 [file Table1.docx]

**Table S1. Information of cattle enrolled in the study.**

|  | **Breeding locations** | **IGRA**  **(IFN-γ release assay)** | ***MTBC* DNA -PCR** |
| --- | --- | --- | --- |
| Healthy_1 | Salerno | NEG | N.P. |
| Healthy_2 | Salerno | NEG | N.P. |
| Healthy_3 | Salerno | NEG | N.P. |
| Healthy_4 | Salerno | NEG | N.P. |
| Healthy_5 | Salerno | NEG | N.P. |
| Healthy_6 | Salerno | NEG | N.P. |
| Healthy_7 | Salerno | NEG | N.P. |
| Healthy_8 | Salerno | NEG | N.P. |
| Healthy_9 | Salerno | NEG | N.P. |
| Healthy_10 | Salerno | NEG | N.P. |
| Healthy_11 | Salerno | NEG | N.P. |
| Healthy_12 | Salerno | NEG | N.P. |
| Healthy_13 | Salerno | NEG | N.P. |
| Healthy_14 | Salerno | NEG | N.P. |
| Healthy_15 | Salerno | NEG | N.P. |
| Healthy_16 | Salerno | NEG | N.P. |
| Healthy_17 | Salerno | NEG | N.P. |
| Healthy_18 | Salerno | NEG | N.P. |
| Healthy_18 | Salerno | NEG | N.P. |
| Infected_1 | Salerno | POS | NEG |
| Infected_2 | Salerno | POS | NEG |
| Infected_3 | Salerno | POS | NEG |
| Infected_4 | Salerno | POS | NEG |
| Infected_5 | Salerno | POS | NEG |
| Infected_6 | Salerno | POS | NEG |
| Infected_7 | Salerno | POS | NEG |
| Infected_8 | Salerno | POS | NEG |
| Infected_9 | Salerno | POS | NEG |
| Infected_10 | Salerno | POS | NEG |
| Infected_11 | Salerno | POS | NEG |
| Infected_12 | Avellino | POS | NEG |
| Infected_13 | Avellino | POS | NEG |
| Infected_14 | Avellino | POS | NEG |
| Infected_15 | Avellino | POS | NEG |
| Infected_16 | Avellino | POS | NEG |
| Infected_17 | Avellino | POS | NEG |
| Affected_1 | Salerno | POS | POS |
| Affected_2 | Salerno | POS | POS |
| Affected_3 | Salerno | POS | POS |
| Affected_4 | Salerno | POS | POS |
| Affected_5 | Salerno | POS | POS |
| Affected_6 | Salerno | POS | POS |
| Affected_7 | Salerno | POS | POS |
| Affected_8 | Salerno | POS | POS |
| Affected_9 | Salerno | POS | POS |
| Affected_10 | Salerno | POS | POS |
| Affected_11 | Salerno | POS | POS |
| Affected_12 | Avellino | POS | POS |
| Affected_13 | Avellino | POS | POS |
| Affected_14 | Avellino | POS | POS |
| Affected_15 | Avellino | POS | POS |
| Affected_16 | Avellino | POS | POS |
| Affected_17 | Avellino | POS | POS |
| Affected_18 | Avellino | POS | POS |
| Affected_19 | Avellino | POS | POS |

MTBC: *Mycobacterium tuberculosis complex*; N.P. Not performed.
